# Correlation Between Clinical Diagnosis, MRI, and Arthroscopy in Diagnosing Shoulder Pathology

**DOI:** 10.7759/cureus.20654

**Published:** 2021-12-23

**Authors:** Aarthi Thiagarajan, Raghu Nagaraj, Kiran Marathe

**Affiliations:** 1 Orthopaedics and Trauma, Hosmat Hospital, Bangalore, IND; 2 Orthopaedics and Trauma, Fortis Hospital, Bangalore, IND

**Keywords:** slap diagnosis, clinical assessment in shoulder, mri in shoulder, diagnostic modalities in shoulder, shoulder pathology

## Abstract

Introduction

Shoulder disorders are frequently encountered by clinicians and are a common cause of musculoskeletal pain in the general population. Clinical tests specific to each shoulder pathology, MRI, and arthroscopy are the most relied upon modalities of diagnosis used by many clinicians. The aim of this study was to correlate clinical tests and MRI with arthroscopy as the gold standard and whether a negative MRI with a positive clinical test could justify an arthroscopy.

Materials and methods

A total of 120 consecutive patients who had a history of shoulder pain or instability were evaluated by clinical tests and MRI, and underwent arthroscopy. Based on the confirmatory findings of arthroscopy, they were classified as True Positive (TP), True Negative (TN), False Positive (FP) and False Negative (FN) for each modality i.e., clinical tests and MRI.

Results

Clinical assessment of rotator cuff tears in comparison to arthroscopy yielded a sensitivity of 96.88%, specificity of 92.86% and diagnostic accuracy of 95%, whilst MRI had a sensitivity of 90.62%, specificity of 92.86% and diagnostic accuracy of 91.67%. In anterior labral lesions, clinical assessment had a sensitivity of 94.44%, specificity of 97.62 % and diagnostic accuracy of 96.67%, whilst MRI had a sensitivity of 83.33%, specificity of 92.86%, with diagnostic accuracy of 90%. Interestingly, in the clinical assessment of superior labral tear from anterior to posterior (SLAP) lesions, a sensitivity of 90%, specificity of 95%, and diagnostic accuracy of 93.33% were observed while MRI had a sensitivity of 60%, specificity of 92.50%, and diagnostic accuracy of 81.67%.

Conclusion

On the basis of these results, clinical assessment appears to be an effective tool in diagnosing shoulder pathologies, whereas MRI, though reliable in the identification of rotator cuff tears and instability, does not identify patients with SLAP lesions effectively. This study reinforces the importance of a good clinical examination of the shoulder, especially in chronic pain and an uncertain MRI, therefore improving patient management.

## Introduction

Shoulder disorders are frequently encountered by clinicians and are the third most common cause of musculoskeletal pain in the general population [1]. Both generic and shoulder specific measurements have shown a significant impact on the health of the affected individual in domains like pain, distress, physical disability to name a few [2,3]. We cannot ignore the ramifications of the impact like monitory loss and the struggle to perform everyday activities, thus enhancing the requirement of accurate diagnosis and precise treatment.

Ideal clinical tests should be repeatable, easy to perform, sensitive, specific, and identify the origin of pathology in the shoulder joint. The accuracy of clinical tests varies according to clinicians as well as patients [4,5]. Moreover, the studies published validating these clinical tests are being questioned for their accuracy and clinical predictability [6,7,8]. Also, the heterogenicity of these studies poses a challenge for meta-analysis [9]. The paucity in the literature is hence a major concern since validation requires an easily accessible standard of reference.

MRI, being non-invasive and highly sensitive in diagnosing shoulder pathology, has a revelatory impact on the diagnosis [10]. Though contraindicated in patients with cardiac pacemakers and ferromagnetic implants, MRI is arguably the most widely used imaging modality in shoulder disorders.

Despite the evolution of MRI in detecting shoulder pathology and given the wide variability in the accuracy of MRI, several lesions are missed. In particular, the accuracy of MRI in the detection of partial rotator cuff tears and tendinitis is low and inconsistent in labral pathology in some centres with no dedicated musculoskeletal radiologists and higher device strength [11,12].

The aim of this study was to correlate the various modalities of diagnosis of shoulder pathology with arthroscopy as the gold standard and to find out whether a negative MRI with a positive clinical test could justify an arthroscopy.

## Materials and methods

The population for this study consisted of 120 consecutive patients from a tertiary care orthopaedic centre with a history of shoulder pain or instability between September 2018 and August 2019. Ethical clearance was obtained for this study from the Hosmat Hospital Ethical Committee.

For the purpose of this study, the shoulder pathologies were organised into (a) Impingement, (b) rotator cuff tears - isolated or with other associations,( c) glenohumeral instability, and {d) glenoid labral lesions further classified as anterior labral (Bankart lesion and associated), superior labral (superior labral tear from anterior to posterior (SLAP) - isolated or associated with biceps tendon abnormalities), and posterior labral. As, osteochondral defects of the humerus and glenoid do not have a popular, validated clinical test, they were not included as an individual entity in the assessment. Osteoarthritis of the glenohumeral and acromioclavicular joint and acromioclavicular joint dislocation were excluded.

Clinical tests of the shoulder joint were performed by shoulder specialists. Hawkins test, modified Neer test, and painful arc test were used to evaluate impingement clinically. Positive apprehension and Jobe Relocation test helped to deduce instability. The tests for SLAP, due to their well-known enigma, were always used as a merger. Amongst the anterior slide test, active compression (O’Brien) test, Speed's test, biceps load tests, compression rotation test, and resisted supination external rotation test, a tailored combination of tests was selected by the shoulder specialists. Rotator cuff tears were identified with the empty can test, inability to abduct, external rotation lag sign, Hornblower’s sign, belly press, and Gerber’s lift-off test. MRI was performed in all patients who were symptomatic after failed conservative management and who were undergoing arthroscopy. A 1.5 T MRI was used and a single musculoskeletal radiologist reported all images. Images were obtained in the axial, coronal oblique, and sagittal oblique planes. The clinical and MRI diagnoses were categorised after the arthroscopy, as described in Table 1.

**Table 1:** Categories based on clinical diagnosis/MRI diagnosis after arthroscopy

Categories	Description
True Positive (TP)	Clinical diagnosis/MRI diagnosis of a pathology, confirmed on arthroscopy
True Negative (TN)	Clinical diagnosis/MRI diagnosis of no pathology, confirmed on arthroscopy
False Positive (FP)	Clinical diagnosis/MRI diagnosis of a pathology, not confirmed on arthroscopy
False Negative (FN)	Clinical diagnosis/MRI diagnosis of no pathology, but pathology confirmed on arthroscopy.

The sensitivity, specificity, positive predictive value (PPV), negative predictive value (NPV), likelihood of a positive test, likelihood of a negative test, and diagnostic accuracy of the clinical tests and MRI for each shoulder pathology was calculated with arthroscopic findings as the gold standard of reference. Data analysis were done using IBM SPSS Statistics for Windows, Version 26.0 (Released 2019, IBM Corp., Armonk, New York).

## Results

The study population was 120 with a mean age of 41 years and a range of 20 to 79 years. There were 73 males and 47 females. In our study, the right side was more commonly affected than the left side. The mechanism of injury was degenerative in 38%, sports-related in 35%, and direct trauma in 27% of the study population.

Rotator cuff tears

In our study, rotator cuff tears were identified arthroscopically in 64 patients, out of which 62 were identified by clinical assessment, showing a sensitivity of 96.88% and specificity of 92.86% and a diagnostic accuracy of 95% (Table 2).

**Table 2:** Clinical assessment of rotator cuff tears in comparison to arthroscopy

Parameter	Estimate	95% CI
Sensitivity	96.88%	83.78 – 99.92
Specificity	92.86%	76.50 – 99.12
Positive predictive value	93.94%	80.28 – 98.33
Negative predictive value	96.30%	79.02 – 99.45
Likelihood ratio of a positive test	13.56	3.56 – 51.64
Likelihood ratio of a negative test	0.03	0.00 – 0.23
Diagnostic accuracy	95%	86.08 – 98.96

Out of 64 rotator cuff tears, 58 were identified by MRI, showing a sensitivity of 90.62% and specificity of 92.86% and a diagnostic accuracy of 91.67% (Table 3).

**Table 3:** MRI of rotator cuff tears in comparison to arthroscopy

Parameter	Estimate	95% CI
Sensitivity	90.62%	74.98 – 98.02
Specificity	92.86%	76.50 – 99.12
Positive predictive value	93.55%	79.15 – 98.23
Negative predictive value	89.66%	74.60 – 96.24
Likelihood ratio of a positive test	12.69	3.32 – 48.46
Likelihood ratio of a negative test	0.10	0.03 – 0.30
Diagnostic accuracy	91.67%	81.61 – 97.24

Bankart lesions

Arthroscopy identified 36 Bankart lesions amongst the unstable shoulders. Of these, clinical assessment identified 34, showing a sensitivity of 94.44%, specificity of 97.62 %, and diagnostic accuracy of 96.67% (Table 4).

**Table 4:** Clinical assessment of Bankart’s lesion in comparison to arthroscopy

Parameter	Estimate	95% CI
Sensitivity	94.44%	72.71- 99.86
Specificity	97.62%	87.43- 99.94
Positive predictive value	94.44%	70.96-99.16
Negative predictive value	97.62%	85.91- 99.64
Likelihood ratio of a positive test	39.68	5.7- 275.9
Likelihood ratio of a negative test	0.06	0.01- 0.38
Diagnostic accuracy	96.67%	88.47-99.59

MRI identified 30 Bankart lesions, showing a sensitivity of 83.33%, specificity of 92.86%, and diagnostic accuracy of 90% (Table 5).

**Table 5:** MRI of Bankart’s lesion in comparison to arthroscopy

Parameter	Estimate	95% CI
Sensitivity	83.33%	58.58-96.42
Specificity	92.86%	80.52-98.50
Positive predictive value	83.33%	62.24-93.82
Negative predictive value	92.86%	82.18-97.34
Likelihood ratio of a positive test	11.67	3.85-35.40
Likelihood ratio of a negative test	0.18	0.06-0.51
Diagnostic accuracy	90%	79.49-96.24

SLAP tears

Superior labral tears were identified in 40 patients arthroscopically, out of which 36 were identified through clinical assessment showing a sensitivity of 90%, specificity of 95%, and diagnostic accuracy of 93.33% (Table 6).

**Table 6:** Clinical assessment of SLAP tears in comparison to arthroscopy

Parameter	Estimate	95% CI
Sensitivity	90%	68.30 – 98.77
Specificity	95%	83.08 – 99.39
Positive predictive value	90%	69.82 – 97.22
Negative predictive value	95%	83.59 – 98.61
Likelihood ratio of a positive test	18	4.63 – 70.04
Likelihood ratio of a negative test	0.11	0.03 – 0.39
Diagnostic accuracy	93.33%	83.80 – 98.15

MRI could identify only 24 out of the 40 SLAP tears identified by arthroscopy, showing a sensitivity of 60%, specificity of 92.50%, and diagnostic accuracy of 81.67% (Table 7).

**Table 7:** MRI of SLAP tears in comparison to arthroscopy

Parameter	Estimate	95% CI
Sensitivity	60%	36.05 – 80.88
Specificity	92.50%	79.61 – 98.43
Positive predictive value	80%	55.99 – 92.64
Negative predictive value	82.22%	72.86 – 88.85
Likelihood ratio of a Positive Test	8	2.54 – 25.16
Likelihood ratio of a Negative Test	0.43	0.25 – 0.75
Diagnostic accuracy	81.67%	69.56 – 90.84

## Discussion

A clinician’s ability to diagnose different shoulder pathologies based on his assessment alone has been questioned by several studies. In instances where either the clinics are busy or the pathology is acute and painful, a thorough clinical assessment is forsaken, and as a matter of course radiological investigations like MRI, magnetic resonance angiogram (MRA) are banked upon. Meta-analysis of three fairly popular clinical tests - Modified Neer test, Hawkins-Kennedy test, and Speed's test, used to examine impingement and SLAP lesion, concluded that none of these tests is diagnostic for their stated pathology [9]. Thus comes the very important question of selecting the diagnostic modality for accurate diagnosis of shoulder pathology and decision-making on surgical intervention.

Rotator cuff tears

In our study, rotator cuff tears were identified arthroscopically in 64 patients. Clinical assessment diagnosed rotator cuff tears in 62 patients, whereas MRI identified 58 out of 64 patients.

Dinnes et al. [13] in their study showed that clinical examination in diagnosing rotator cuff tears had sensitivity of 90% but specificity of 54%, while MRI in diagnosing rotator cuff tears had sensitivity of 89% and specificity of 93%, which is comparable to our study. Another study by Torensten et al. [12] showed identification of rotator cuff tears by MRI with a sensitivity of 96%. Fukuda et al. [14] described that there is a 7.2% incidence of purely intra-tendinous supraspinatus tear. This is paramount in deducing the accuracy of MRI, which identifies the presence of intra- substance rotator cuff tears but are missed arthroscopically, thus falsely reducing the accuracy of MRI if the tears do not communicate with either the subacromial space or glenohumeral joint. Vice versa, surgical interventions based on intrasubstance tears would not be beneficial for the patient. MRI unarguably identified rotator cuff tears effectively in our study; however, its inability to clearly differentiate partial and complete tears places a surgeon, who is relying on MRI, in a crux to take a surgical decision upon intervention.

Bankart tears

Amongst the clinically unstable shoulders, Bankart lesion was found arthroscopically in 36 patients. Clinical examination picked up the pathology in 34 patients and MRI picked it up in 30. In their study, Loh et al. [15] showed that clinical examination diagnosed Bankart lesion with a sensitivity of 94% while MRI had 89% sensitivity, which was comparable to our study. Although Green et al. [16] concluded in their study that MRI is not useful in anterior shoulder instability, we had a very high diagnostic accuracy in our study.

SLAP tears

A total of 40 patients were known to have SLAP tears through arthroscopy. Clinical examination helped to pick 36 of them, while MRI identified only 24. In their study, Liu et al. [17] recognised that clinical examination yielded a sensitivity of 90% and specificity of 85%, and MRI yielded a sensitivity of 59% and specificity of 85%, which is comparable to our study. Unlike the evaluation of other shoulder disorders, identification of SLAP lesions by MRI was poor owing to reduced sensitivity and diagnostic accuracy. Nevertheless, studies do show that provocative manoeuvres like longitudinal traction of the arm or positioning the shoulder in abduction and external rotation during imaging or additional planes parallel to the biceps tendon improve the diagnostic accuracy of MRI in SLAP tears [18,19,20].

Several studies have identified the sensitivity of MRA in detecting glenoid labral lesions, but we need to bear in mind the financial burden on the patient and the rarer complications like contrast allergy, extravasation of contrast, and prolonged waiting time for the investigation thus delaying the definite management of the patient. Moreover, a majority of studies recommending MRA identified the patient group at the time of arthroscopy, retrospectively evaluating those with proven labral lesions and so, questioning the results. Thus, the selection of operative candidates or assistance in planning the surgical procedure in SLAP lesions would demand a tailormade clinical assessment of a symptomatic patient. We cannot ignore the fact that clinical tests in our study were done by shoulder specialists and their knowledge and experience could have improved our results in comparison to studies where clinical assessments were performed by non-specialist orthopaedic surgeons.

## Conclusions

On the basis of these results, clinical assessment appears to be an effective tool in diagnosing shoulder pathologies, whereas MRI, though reliable in the identification of rotator cuff tears and instability, does not identify patients with SLAP lesions effectively. We recommend going ahead with surgical treatment of patients who have been clinically identified to have a slap tear, in spite of a negative MRI, to provide better results after a sufficient period of failed conservative treatment. Considering that clinical examination has shown higher sensitivity and specificity in all the shoulder conditions, a good clinical examination will not miss pathology in most patients. This study reinforces the importance of a good clinical examination of the shoulder, especially in chronic pain and an uncertain MRI, and warrants the need for an arthroscopy, therefore improving patient management.
